# A Smaller Tibiotalar Sector Is a Risk Factor for Recurrent Anterolateral Ankle Instability after a Modified Broström-Gould Procedure

**DOI:** 10.1177/10711007241227925

**Published:** 2024-02-23

**Authors:** Flamur Zendeli, Patrick Pflüger, Arnd F. Viehöfer, Sandro Hodel, Stephan H. Wirth, Mazda Farshad, Lizzy Weigelt

**Affiliations:** 1 Department of Orthopedics, Balgrist University Hospital, University of Zurich, Zurich, Switzerland

**Keywords:** chronic lateral ankle instability, recurrent ankle instability, lateral ligament repair, tibiotalar sector

## Abstract

**Background:**

Several demographic and clinical risk factors for recurrent ankle instability have been described. The main objective of this study was to investigate the potential influence of morphologic characteristics of the ankle joint on the occurrence of recurrent instability and the functional outcomes following a modified Broström-Gould procedure for chronic lateral ankle instability.

**Methods:**

Fifty-eight ankles from 58 patients (28 males and 30 females) undergoing a modified Broström-Gould procedure for chronic lateral ankle instability between January 2014 and July 2021 were available for clinical and radiological evaluation. Based on the preoperative radiographs, the following radiographic parameters were measured: talar width (TW), tibial anterior surface (TAS) angle, talar height (TH), talar radius (TR), tibiotalar sector (TTS), and tibial lateral surface (TLS) angle. The history of recurrent ankle instability and the functional outcome using the Karlsson Score were assessed after a minimum follow-up of 2 years.

**Results:**

Recurrent ankle instability was reported in 14 patients (24%). The TTS was significantly lower in patients with recurrent ankle instability (69.8 degrees vs 79.3 degrees) (*P* < .00001). The multivariate logistic regression model confirmed the TTS as an independent risk factor for recurrent ankle instability (OR = 1.64) (*P* = .003). The receiver operating characteristic curve analysis revealed that patients with a TTS lower than 72 degrees (=low-TTS group) had an 82-fold increased risk for recurrent ankle instability (*P* = .001). The low-TTS group showed a significantly higher rate of recurrent instability (58% vs 8%; *P* = .0001) and a significantly lower Karlsson score (65 points vs 85 points; *P* < .00001).

**Conclusion:**

A smaller TTS was found to be an independent risk factor for recurrent ankle instability and led to poorer functional outcomes after a modified Broström-Gould procedure.

**Level of Evidence:**

Level IV, retrospective cohort study.

## Graphical abstract

**Graphical Abstract:**
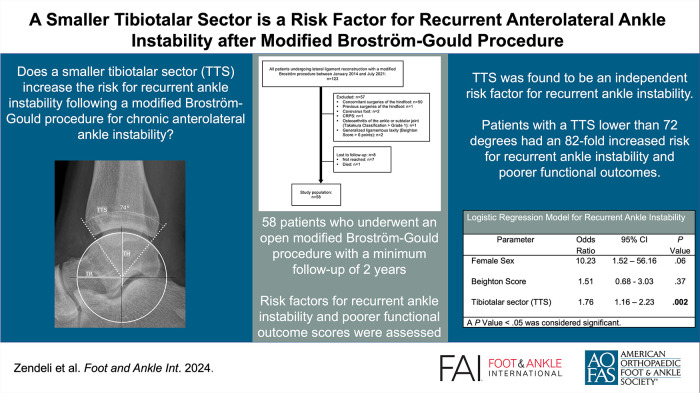
This is a visual representation of the abstract.

## Introduction

Ankle sprains are among the most common ankle injuries.^9,15,20^ The majority of ankle sprains recover with nonoperative management. Yet, in about 20% of the cases, recurrent ankle sprains lead to symptomatic chronic ankle instability (CAI), which requires surgery.^15,21,25^

The modified Broström-Gould procedure is considered an effective and anatomical repair of the lateral ligaments aiming to restore ankle stability and enhance functional outcomes.^4,5,35,40^ However, recurrent ankle instability after primary ligament repair has been reported in 18% to 28% of the patients.^29,30,53-55^

Potential clinical risk factors for recurrent ankle instability after lateral ligament repair are generalized ligamentous laxity and poor quality of the anterior talofibular ligament remnant, which also lead to poorer clinical outcomes.^34,52,53^

Several morphologic risk factors of the ankle joint have been related to CAI such as a hindfoot varus,^6,28,43^ a varus tilt of the tibial plafond,^44^ a larger talar radius and talar height, and a smaller tibiotalar sector.^12^ However, little is known if these factors also play a role in recurrent ankle instability after lateral ligament repair.^54,55^

The objective of this study was to investigate the potential association between an unstable morphologic configuration of the ankle joint and the occurrence of recurrent instability and the influence of these morphologic factors on the clinical outcome after a modified Broström-Gould procedure for CAI. We hypothesized that a smaller tibiotalar sector, a varus tilt of the tibial plafond, a larger talar radius, and a higher talus, a smaller talar width, and a smaller ankle mortise angle are risk factors for recurrent ankle instability and are associated with a poorer clinical outcome.

## Materials and Methods

### Patient Selection

This retrospective single-center study was approved by the local ethics committee (BASEC-Nr. 2021-0009). All patients who had undergone reconstruction of the lateral ligaments with an open modified Broström-Gould procedure between January 2014 and July 2021 were enrolled in the study by searching our hospital’s own patient archive. Indication for surgery was a patient history of recurrent ankle sprains with a positive anterior drawer test of the talus in the clinical examination and failure of nonoperative management for at least 6 months. The study period was chosen to ensure a minimum clinical follow-up of 2 years. Exclusion criteria were (1) neurologic disorders, (2) previous surgeries of the hindfoot, (3) concomitant surgeries of the hindfoot (eg, calcaneal osteotomy, peroneal tendon repair), (4) other lateral ligament reconstruction techniques (eg, anatomical ligament reconstruction), (5) cavovarus foot (defined as Meary angle >4 degrees, anteroposterior talocalcaneal angle >20 degrees or clinical documentation in the patient’s archive), (6) generalized ligamentous laxity (defined as a Beighton score ≥4 points^19,38^), (7) ankle osteoarthritis, grade 2 or higher according to the Takakura classification.^45,46^

**Figure 1.**
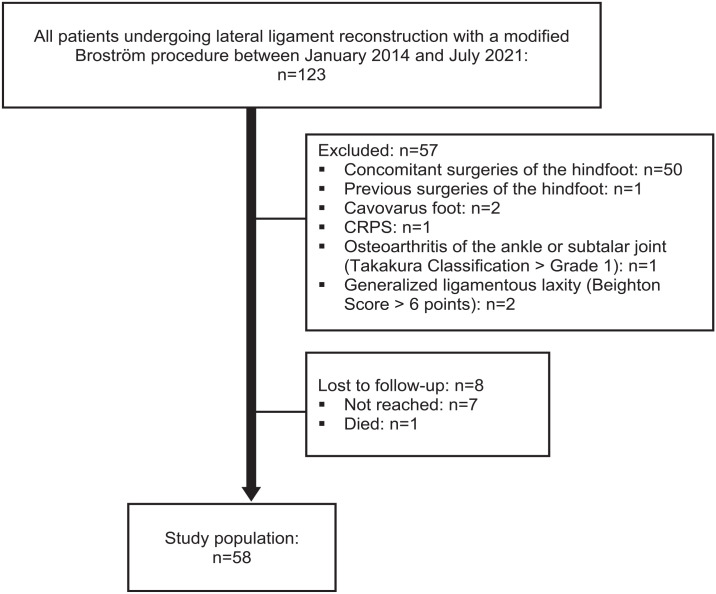
Patient flow diagram.

Based on the exclusion criteria, 57 of the 123 eligible patients (123 ankles) were excluded (Figure 1). Another 8 patients were lost to follow-up, of which 1 patient died 2 years after surgery because of an underlying oncologic disease. Finally, 58 patients (28 males and 30 females; 58 ankles, in the following referred to as cases) were available for clinical evaluation after a median follow-up of 4.3 (IQR, 2.5-6.1) years. The median age at the time of surgery was 30 (IQR, 22-45) years.

### Clinical Assessment

All study participants were contacted via mail or REDCap electronic data capture system.^13,14^ Demographic data (age, sex, body mass index [BMI]) and smoking status were recorded. Recurrent ankle stability was chosen as the primary outcome parameter, defined by another ankle sprain after the Broström repair. We distinguished between low-velocity and high-velocity sprains in our study. Low-velocity sprains were defined as minor traumas such as twisting the ankle while walking on even ground. High-velocity sprains were characterized by significant trauma such as ankle sprains on slippery ground or while descending stairs.

The functional outcome was assessed as a secondary outcome parameter based on the modified Karlsson Score (0-90 points).^17,18^ The score contains the following 8 subcategories: pain (20), swelling (10), subjective instability (15), stiffness (5), stair climbing (10), running (10), work activities (15), use of a support device (5).

### Radiographic Assessment

**Figure 2.**
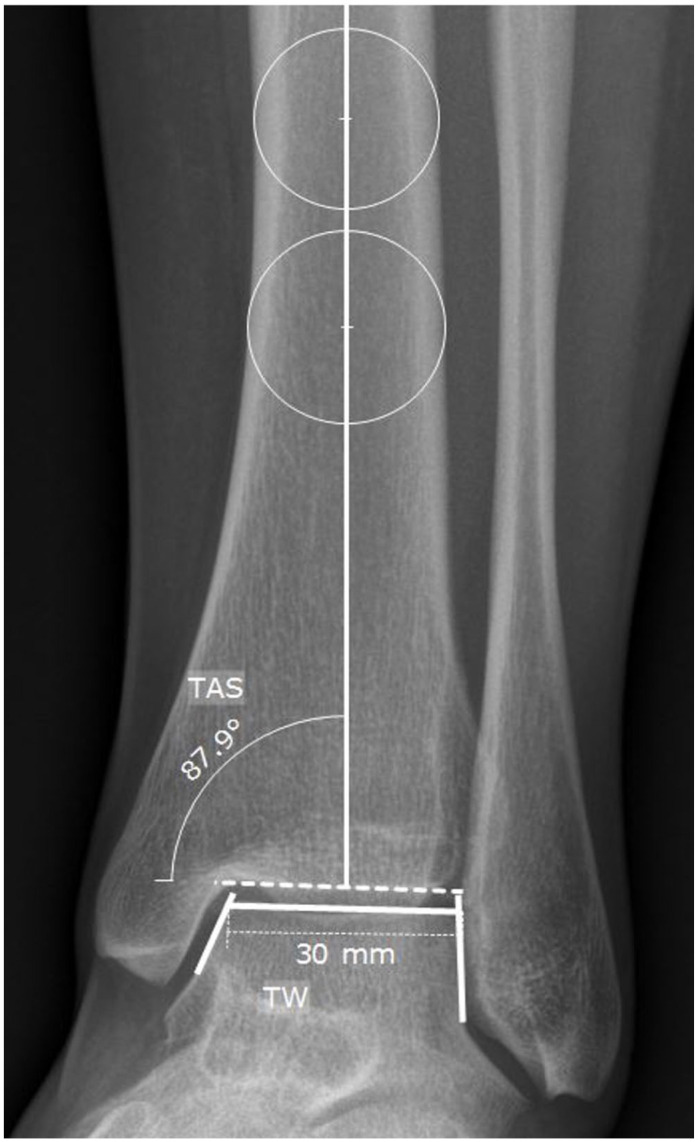
Measurements of tibial anterior surface (TAS) angle and talar width (TW) on an anteroposterior weightbearing radiograph: the TAS angle is the angle between the tibial axis and distal tibial articular joint surface measured in degrees. For the TW, a tangent line is drawn along the medial and lateral talar shoulder. Another tangent line is drawn on the joint surface of the talar dome. The TW is the distance of the 2 intersections of these lines, measured in millimeters.

**Figure 3.**
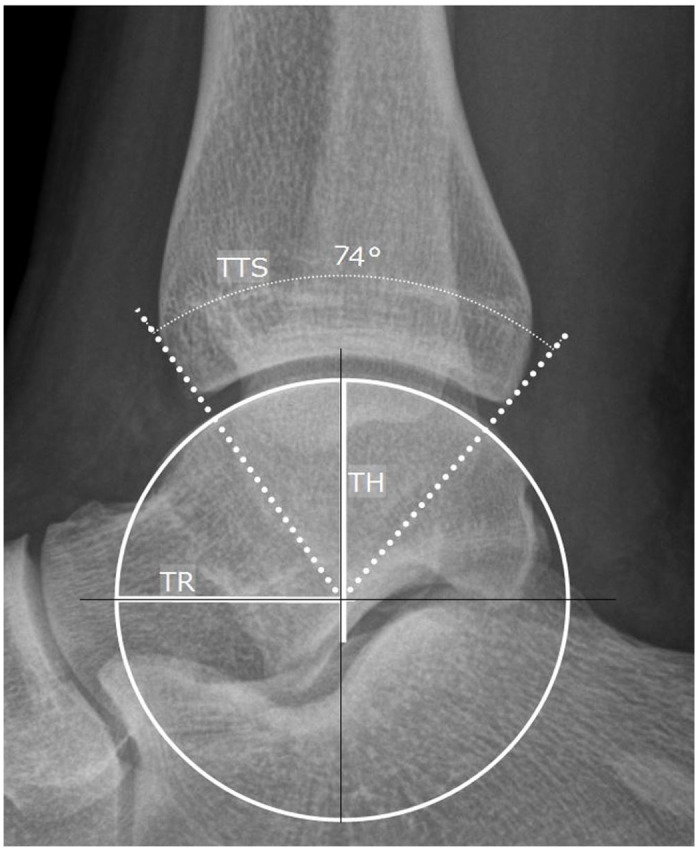
Measurements of the talar radius (TR), talar height (TH), and tibiotalar sector (TTS) on a lateral weightbearing radiograph: the center of the talus was identified by measuring the most appropriate circle on the articular surface of the talar dome. The TR is the straight line from the center of the talus to the circumference of the circle, measured in millimeters. A vertical line to the floor was drawn through the center of the talus. The TH is given by the distance of the vertical line intersecting the articular surface of the talar dome and inferior surface of the talar body, measured in millimeters. From the center of the talus a line was set to the anterior and posterior borders of the distal tibia. The angle formed by these 2 lines represents the TTS, quantifying the extent of tibial coverage over the talus, measured in degrees.

**Figure 4.**
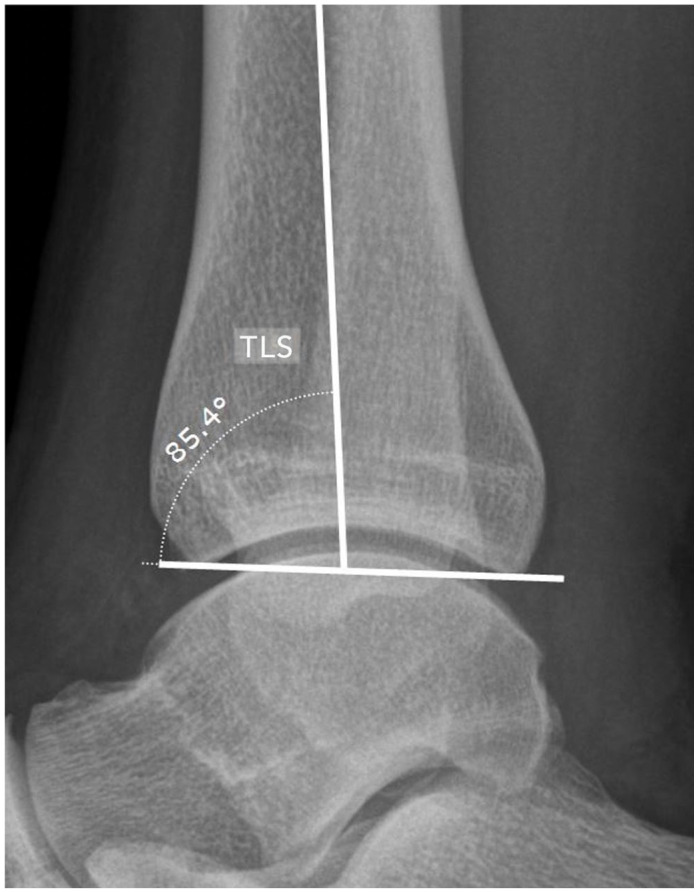
Measurements of the tibial lateral surface (TLS) angle on a lateral weightbearing radiograph: The TLS angle is the angle between the tibial shaft axis and a line extending from the anterior border to the posterior border of the distal tibia, measured in degrees.

Based on the preoperative anteroposterior and lateral weightbearing ankle radiographs, the following measurements were carried out using the mediCAD planning software (mediCAD Hectec GmbH, Altdorf, Germany): talar width (TW), tibial anterior surface (TAS) angle, talar height (TH), talar radius (TR), tibiotalar sector (TTS), and tibial lateral surface (TLS) angle (Figures 2-4).^12,16,31,42,45^ All radiographic parameters were measured by 2 independent investigators not involved in the initial surgical treatment and blinded to the patient’s clinical history and outcome. The measurements of TW, TH, and TR were divided by the body height to adjust for sex- and body height–related differences.^2^

### Surgical Technique

An open modified Broström-Gould procedure was performed in all cases, with the patient in a lateral decubitus position. A curvilinear skin incision was centered over the distal anterior border of the lateral malleolus. The periosteum of the distal fibula was incised in line with the skin incision and raised anteriorly toward the lateral gutter of the ankle. The proximal border of the extensor retinaculum was mobilized for later augmentation to the distal fibula. The ATFL remnant was identified and sharply elevated from its footprint at the anterior tip of the fibula. The CFL was inspected and only released in case of insufficient tension. The ligamentous quality in terms of thickness and mechanical resistance was assessed to decide if a modified Broström-Gould procedure was feasible. If a repair was impossible, an augmented reconstruction procedure was performed with a gracilis allograft tendon. Otherwise, 2 all-suture anchors (JuggerKnot Soft Anchor System; Zimmer Biomet, Warsaw, IN) were placed in the anatomical footprint ATFL. The AFTL was reattached with mattress sutures holding the foot in 0 degrees of dorsiflexion and slight eversion. In case of CFL insufficiency, the second anchor was placed more distally. The extensor retinaculum was sutured to the periosteum with a 2-0 monofilament running suture (PROLENE polypropylene; Ethicon, Johnson & Johnson, New Brunswick, NJ).

### Postoperative Management

After 6 weeks of partial weightbearing in a lower leg cast, patients commenced physical therapy involving ankle mobilization, proprioception, and strength training. The ankle was protected for outdoor activities for another 6 weeks.

### Statistical Analysis and Data Collection

Continuous variables not meeting the criteria for normality were assessed with the Mann-Whitney *U* test and reported as median and interquartile ranges (IQRs). Categorical and qualitative variables were evaluated with the chi-square test (or Fisher exact test if n < 5) and presented as numbers and percentages. A *P* value <.05 was considered significant. All significant measurement differences were included in a multivariate logistic regression model. A post hoc Bonferroni correction was used to adjust for multiple comparisons. A *P* value <.01 was considered significant. A receiver operating characteristic curve analysis was calculated based on the Youden index to determine the threshold values for significant measurement values. Intra- and interobserver reliabilities of the radiographic measurements were assessed using intraclass correlation coefficients (ICCs) along with their corresponding 95% CIs. The interpretation of ICC values was as follows: below 0.50, poor agreement; between 0.50 and 0.75, moderate agreement; between 0.75 and 0.90, good agreement; above 0.90, excellent agreement.^22,39^ According to a post hoc power analysis comparing the TTS between patients with and without recurrent ankle instability, the statistical power was greater than 0.8.

Statistical analysis was performed using R Studio software (R Foundation for Statistical Computing, Vienna, Austria).

## Results

After a median follow-up period of 4.3 (IQR, 2.5-6.1) years, 14 patients (24%) reported recurrent ankle instability with a median of 4 (IQR, 3-5) episodes of recurrent ankle sprains. Only 1 patient reported a high-velocity sprain after the Brostöm repair, followed by 4 low-velocity sprains. We decided to include this patient in the recurrent ankle instability group.

Three of the 14 patients (21%) with recurrent ankle instability needed revision surgery. Their median TTS was 69 (range, 68-71) degrees. All of them underwent anatomical lateral ligament reconstruction using a gracilis allograft tendon after a median of 28 (range, 26-29) months after the initial surgery. One of 3 patients (TTS 69 degrees) needed a second revision surgery due to recurrent ankle instability, 30 months following anatomical reconstruction. The other 2 patients remained stable (follow-up 14 and 28 months, respectively).

In the univariate analyses, female sex (86% vs 41%; *P* = .005) and a higher Beighton score (2 points vs 0 points; *P* = .001) were significantly associated with recurrent ankle instability. The follow-up period, BMI, body height, age at surgery, and smoking status showed no significant differences (Table 1). The median Karlsson Score was significantly lower in patients with recurrent ankle instability (74 points vs 87 points; *P* = .02; Table 2).

**Table 1.** Demographic Parameters in Patients With and Without Recurrent Ankle Stability.

Parameter	With Recurrence (n=14; 24%)	Without Recurrence (n=44; 76%)	*P* Value^a^
Follow-up period (y)^b^	4.5 (3.7-7.1)	4.2 (2.4-5.7)	.22
Reoperation^c^	3 (21)	0	–
Sex			.005
Female	12 (86)	18 (41)	
Male	2 (14)	26 (59)	
BMI^b^	26 (21-32)	26 (24-29)	.98
Age (y)^b^	31 (21-43)	30 (26-47)	.35
Smoker^c^	4 (29)	16 (36)	.75
Beighton score (points)^b^	2 (1-3)	0 (0-1)	**.001**

Abbreviation: BMI, body mass index.

a A *P* value <.05 was considered significant (shown in bold).

b Values are expressed as the median with the interquartile range (q1-q3) in parentheses. The Mann-Whitney *U* test was used to analyze the differences of the demographic parameters for patients with and without recurrent ankle stability.

c Values are expressed as absolute numbers with percentages in parentheses. The Fisher exact test was used to analyze the difference.

**Table 2.** Functional Outcome in Patients With and Without Recurrent Ankle Stability.

Parameter	With Recurrence (n=14; 24%)	Without Recurrence (n=44; 76%)	*P* Value^a^
Karlsson score (points)^b^	74 (43-85)	87 (79-90)	**.02**

a A *P* value <.05 was considered significant (shown in bold).

b Values are expressed as the median, with the interquartile range (q1-q3) in parentheses. The Mann-Whitney *U* test was used to analyze the differences of the functional outcome for patients with and without recurrent ankle stability.

Patients with recurrent ankle instability showed a significantly smaller TW (and TW–body height ratio), TR (and TR–body height ratio), and TTS compared to patients with stable ankles. All other radiographic parameters showed no significant differences between the 2 groups (Table 3).

**Table 3.** Radiographic Parameters in Patients With and Without Recurrent Ankle Instability.

Parameter	With Recurrence (n=14; 24%)	Without Recurrence (n=44; 76%)	*P* Value^a^
Talar width (TW) (mm)^b^	27.5 (26-28.8)	30 (27.3-33.0)	**.01**
TW–body height ratio (10^–3^)^b^	16.3 (15.5-16.7)	17.5 (16.4-18.3)	**.02**
Talar height (TH) (mm)^b^	27.0 (26.0-28.0)	30.0 (26.0-31.0)	.10
TH–body height ratio (10^–3^)^b^	16.0 (15.3-17.0)	16.9 (15.3-18.2)	.31
Talar radius (TR) (mm) ^b^	19.5 (18.3-20.8)	22.0 (20.0-24.5)	**.02**
TR–body height ratio (10^–3^)^b^	12.0 (11.0-12.2)	12.6 (11.8-13.7)	**.03**
Tibial anterior surface (TAS) angle (degrees)^b^	88.5 (86.1-89.7)	89.2 (87.9-91.1)	.11
Tibiotalar sector (TTS) (degrees)^b^	69.8 (68.7-72.0)	79.3 (74.8-81.4)	**<.00001**
Tibial lateral surface (TLS) angle (degrees)^b^	85.1 (84.0-86.4)	84.9 (86.3-83.0)	.53

a A *P* value <.05 was considered significant (shown in bold).

b Values are expressed as the median with the interquartile range (q1-q3) in parentheses. The Mann-Whitney *U* test was used to analyze the differences of the radiographic parameters for patients with and without recurrent ankle stability.

The multivariate logistic regression model confirmed the TTS as an independent risk factor for recurrent ankle instability (OR = 1.64; *P* = .003; Table 4). Eleven of 19 patients (58%) with a TTS lower than 72 degrees (=low-TTS group) showed recurrent ankle instability compared to 3 of 39 patients (8%) with a TTS equal to or higher than 72 degrees (high-TTS group) (*P* = .0001). The receiver operating characteristic curve analysis revealed that patients in the low-TTS group had an 82-fold increased risk for recurrent ankle instability (area under the curve = 0.91, *P* = .001). The median Karlsson Score was significantly lower in the low-TTS group (65 points; IQR, 41-72) compared with the high-TTS group (85 points; IQR, 77-90; *P* < .00001). The inter- and intraobserver reliabilities were excellent for all radiographic parameters (Supplementary Table S1).

**Table 4.** Logistic Regression Model for Recurrent Ankle Instability.^a^

Parameter	Odds Ratio	95% CI	*P* Value^a^
Female sex	3.2	0.6-20.3	.56
Beighton score	1.42	0.6-3.37	.43
Tarsal width–body height ratio	1.42	0.69-2.89	.34
Talar radius–body height ratio	1.11	0.53-2.33	.78
Tibiotalar sector (TTS)	1.64	1.19-2.26	**.003**

a The Bonferroni correction was used to adjust for multiple comparisons. A *P* Value < .01 was considered significant (shown in bold).

## Discussion

Although primary lateral ligament repair for CAI is a standard procedure, there is still a substantial number of patients who suffer at least 1 recurrent ankle sprain.^1,29,53-55^ The assessment of risk factors for recurrent ankle instability seems essential in the context of optimizing lateral ligament repair as CAI could result in the development of posttraumatic ligamentous ankle osteoarthritis.^48^ Several clinical risk factors for recurrent ankle instability have been described.^34,52-55^ However, to our knowledge, there is no study investigating the influence of ankle morphology on the recurrence of ankle instability after a modified Broström-Gould procedure.

In a biomechanical model,^11^ it was observed that as the TTS angle decreases, the force required to dislocate the tibia reduces. This observation led to the hypothesis that ankle joints characterized by a smaller TTS may be less stable. In this study, we confirmed that patients with recurrent ankle instability after a modified Broström procedure showed a significantly lower TTS angle. The multivariate logistic regression model revealed the TTS as an independent risk factor for recurrent ankle instability. Patients with a low TTS (<72 degrees) had an 82-fold higher risk of recurrent ankle instability and a significantly poorer Karlsson score than patients with a high TTS (≥72 degrees).

Frigg et al^12^ compared the TTS, talar height, and talar radius in patients with CAI to a control group of healthy subjects. The talar radius in patients with CAI was significantly larger compared to the control group, whereas the TTS angle was significantly lower (*P* < .001). They explained their findings in a way that a larger talar radius leads to a flatter talar dome, which withholds less restraint in the tibia and thus represents a risk factor for CAI. In contrast, we found a smaller talar radius and a smaller TTS in patients with recurrent ankle instability, which we attribute to a smaller tibiotalar containment. The major limitation of Frigg’s study was that the measurements were not referenced to the body height of the patients. This could have potentially introduced a measurement bias, as patients with a larger body height apparently also have a larger talar width and radius.

Yoshimoto et al^54^ investigated 57 patients who underwent arthroscopic lateral ligament reconstruction and showed that a varus-tilted tibial plafond was associated with recurrent ankle instability. Patients with a TAS angle <86.2 degrees had a significantly higher frequency of recurrent ankle instability than patients with a TAS angle above this threshold (43.8% vs 7.3%) (*P* = .0031). In our study, the median TAS angle was also lower in patients with recurrent ankle instability (88.5 degrees vs 89.2 degrees) but without significant difference (*P* = .11). One reason for the different results may have been the inclusion criteria. Yoshimoto did not record the patients’ Beighton scores and hindfoot axis, so patients with general ligamentous laxity or cavovarus foot may have influenced the recurrence rates. It has been shown that patients with generalized ligamentous laxity (defined as a Beighton score ≥4 points) and cavovarus foot have a significantly higher risk of recurrent ankle instability and inferior clinical outcomes.^7,34,36,37,52,55^ We therefore deliberately excluded patients with these confounding factors, which is a strength of our study. Furthermore, the average follow-up of our study was longer (1.4 years vs 4.3 years) and the overall recurrence rate was lower (14% vs. 17.5%) than in Yoshimoto’s study.

The rate of female sex was significantly higher in patients with recurrent ankle instability in our study population (86% vs 41%). However, female sex was not an independent risk factor according to our multivariate logistic regression (OR = 3.2; *P* = .56). Yoshimoto et al^54^ also found a high rate of 90% of female patients with recurrent ankle instability after arthroscopic lateral ligament repair compared to 40% male patients (*P* = .0049). This was also confirmed in another study^29^ focusing on clinical risk factors for recurrent instability after arthroscopic lateral ligament repair (81% vs 43%). However, other studies showed no correlation between female sex and recurrent ankle instability.^34,52,55^

This study has several limitations. First, because of the retrospective design, the study lacks preoperative Karlsson scores. However, the postoperative Karlsson score is similar to other studies that investigated the clinical outcome of lateral ligament repair for chronic ankle instability.^27,29,34,47^ Furthermore, the rate of revision surgery after primary lateral ligament repair in our study population (5%) and the rate of recurrent ankle instability (24%) was comparable to previous studies.^23,30,54,55^ It should be noted that there is no universally accepted definition of recurrent ankle instability, which can pose challenges when comparing results.^29,34,35,54^ Especially the one patient in our study group who experienced a high-velocity trauma after Broström repair may have remained stable without the second major trauma. Second, all investigators measuring the outcome variables were orthopaedic specialists or orthopaedic surgeons and not radiologists. However, both intra- and interobserver reliabilities were excellent for all measured values. Third, the successful healing of the lateral ligament reconstruction relies not only on the osseous ankle configuration but also on the influence of the surgeons’ skills, the stability of the fixation, and other biological factors. A previous study showed that the recurrence of ankle instability after arthroscopic lateral ligament reconstruction can be attributed to poor ATFL remnant quality.^53^ Furthermore, a neglected CFL injury can also lead to recurrent ankle instability.^29,30^ Based on the patients’ medical records, we were not able to adjust for these possible confounders. Fourth, the surgical interventions were performed by 5 different orthopaedic consultant surgeons, which may have introduced a bias concerning the operative technique that was applied. However, all surgeons were trained by the head of the foot and ankle team to perform the surgery in a standardized manner and had at least 1-year experience in foot and ankle surgery. Moreover, the cases in which recurrent ankle instability occurred were evenly distributed among the treating surgeons. Lastly, we referenced our measurements to the body height to adjust for sex- and body height–related differences. However, Astolfi et al^2^ only found a moderate correlation between the body height and the measures of the distal tibia and talus. We still think that reporting the ratios is more accurate than the simple measurements.

Despite these limitations, we think that our study adds further important knowledge to potential risk factors for recurrent ankle instability. Patients presenting with a small TTS should be advised of their potentially higher failure rate after a modified Broström-Gould procedure. To date, we have no data to make any recommendations on whether augmented ankle reconstruction techniques should be preferred in these cases. In our study, all 3 patients who underwent revision surgery had a small TTS below 72 degrees. One of the 3 needed another revision; the others remained stable. Larger cases series are needed to investigate the TTS as a risk factor after primary allograft reconstruction or augmented repair. Even though allograft reconstructions and augmented repairs demonstrated good results compared to the Broström procedure in terms of clinical outcomes, the data are highly heterogeneous and long-term studies are missing.^26,32,33,41,51^ It should also be noted that the use of augmented reconstructions and allograft reconstructions is associated with higher material costs, and a reduced range of motion has been reported in some cases.^8,10,24^ For now, we therefore preserve augmented reconstructions for revision surgery and for cases in which a modified Broström procedure is not feasible.

A smaller TTS as a risk factor for recurrent ankle instability may have also a clinical implication when treating patients with CAI and concomitant anterior ankle impingement. Removing tibial osteophytes as recommended in the literature^3,49,50^ could decrease the tibial coverage of the talus, respectively, the TTS angle. This could potentially predispose these patients to recurrent ankle instability. Consequently, the removal of tibial osteophytes may warrant careful consideration, particularly in patients with a small TTS angle.

## Conclusion

A smaller TTS was associated with recurrent ankle instability and poorer functional outcomes after a modified Broström-Gould procedure

## Supplemental Material

sj-docx-2-fai-10.1177_10711007241227925 – Supplemental material for A Smaller Tibiotalar Sector Is a Risk Factor for Recurrent Anterolateral Ankle Instability after a Modified Broström-Gould Procedure Supplemental material, sj-docx-2-fai-10.1177_10711007241227925 for A Smaller Tibiotalar Sector Is a Risk Factor for Recurrent Anterolateral Ankle Instability after a Modified Broström-Gould Procedure by Flamur Zendeli, Patrick Pflüger, Arnd F. Viehöfer, Sandro Hodel, Stephan H. Wirth, Mazda Farshad and Lizzy Weigelt in Foot & Ankle International

sj-pdf-1-fai-10.1177_10711007241227925 – Supplemental material for A Smaller Tibiotalar Sector Is a Risk Factor for Recurrent Anterolateral Ankle Instability after a Modified Broström-Gould Procedure Supplemental material, sj-pdf-1-fai-10.1177_10711007241227925 for A Smaller Tibiotalar Sector Is a Risk Factor for Recurrent Anterolateral Ankle Instability after a Modified Broström-Gould Procedure by Flamur Zendeli, Patrick Pflüger, Arnd F. Viehöfer, Sandro Hodel, Stephan H. Wirth, Mazda Farshad and Lizzy Weigelt in Foot & Ankle International
