# Supplementary material for: A Smaller Tibiotalar Sector Is a Risk Factor for Recurrent Anterolateral Ankle Instability after a Modified Broström-Gould Procedure
Source: Foot Ankle Int. 2024 Feb 23;45(4):338–47. doi: 10.1177/10711007241227925 (PMC11192177; doi:10.1177/10711007241227925)
Supplement: sj-docx-2-fai-10.1177_10711007241227925 – Supplemental material for A Smaller Tibiotalar Sector Is a Risk Factor for Recurrent Anterolateral Ankle Instability after a Modified Broström-Gould Procedure [file sj-docx-2-fai-10.1177_10711007241227925.docx]

| TABLE 5 Supp. Inter- and intra-observer reliabilities for radiographic parameters | | | |
| --- | --- | --- | --- |
| Parameter | ICC | 95% CI | *P* Value |
| Inter-observer reliability |  |  |  |
| Tarsal width (TW) | 0.97 | [0.94;0.98] | **<0.0001** |
| ﻿Tibial anterior surface (TAS) angle | 0.98 | [0.97;0.99] | **<0.0001** |
| Talar height (TH) | 0.94 | [0.89; 0.96] | **<0.0001** |
| Talar radius (TR) | 0.94 | [0.90; 0.97] | **<0.0001** |
| Tibiotalar sector (TTS) | 0.98 | [0.96; 0.99] | **<0.0001** |
| Tibial lateral surface (TLS) angle | 0.97 | [0.95; 0.98] | **<0.0001** |
| Intra-observer reliability |  |  |  |
| Tarsal width (TW) |  |  |  |
| Observer 1 | 0.98 | [0.96; 0.99] | **<0.0001** |
| Observer 2 | 0.92 | [0.87; 0.95] | **<0.0001** |
| Tibial anterior surface (TAS) angle |  |  |  |
| Observer 1 | 0.97 | [0.93; 0.98] | **<0.0001** |
| Observer 2 | 0.95 | [0.89; 0.98] | **<0.0001** |
| Talar height (TH) |  |  |  |
| Observer 1 | 0.95 | [0.91; 0.97] | **<0.0001** |
| Observer 2 | 0.97 | [0.95; 0.98] | **<0.0001** |
| Talar radius (TR) |  |  |  |
| Observer 1 | 0.96 | [0.93; 0.97] | **<0.0001** |
| Observer 2 | 0.95 | [0.91; 0.97] | **<0.0001** |
| Tibiotalar sector (TTS) |  |  |  |
| Observer 1 | 0.98 | [0.97; 0.99] | **<0.0001** |
| Observer 2 | 0.98 | [0.96; 0.99] | **<0.0001** |
| Tibial lateral surface (TLS) angle |  |  |  |
| Observer 1 | 0.91 | [0.85; 0.95] | **<0.0001** |
| Observer 2 | 0.96 | [0.93; 0.98] | **<0.0001** |
| ICC: Intra-class correlation coefficient  CI: Confidence interval | | | |
| A *P* Value < .05 was considered significant. | | | |
